# An actinomycosis infection resembling peritoneal dissemination of rectal cancer: a case report

**DOI:** 10.1186/s40792-024-02005-6

**Published:** 2024-09-06

**Authors:** Yukiko Fukunaga, Hiromichi Maeda, Sachi Yamaguchi, Miho Tsutsui, Ken Okamoto, Tomoki Tanaka, Masahiro Maeda, Akira Marui, Tsutomu Namikawa, Michiya Kobayashi, Satoru Seo

**Affiliations:** 1 Department of Surgery, Kochi Medical School, Kochi University, Kohasu, Oko-Cho, Nankoku, Kochi, 783-8505 Japan; 2 Cancer Treatment Center, Kochi Medical School Hospital, Nankoku, Japan; 3 Department of Diagnostic Pathology, Kochi Medical School Hospital, Nankoku, Japan

**Keywords:** Peritoneal dissemination, Mesh, Hernia, Abdominal actinomycosis

## Abstract

**Background:**

Actinomycosis is a suppurative and granulomatous inflammation commonly caused by *Actinomyces israelii*. Due to its rarity and the paucity of characteristic clinical features, diagnosis of intra-abdominal actinomycosis is challenging, especially when the patient has a treatment history of abdominal cancer.

**Case presentation:**

The patient is a 72-year-old man who has a history of multiple abdominal surgeries for rectal cancer, including low anterior resection for primary rectal cancer, partial hepatic resection for metachronous liver metastasis, and Hartmann surgery for local recurrence. The patient has also undergone parastomal hernia repair using the Sugarbaker method. One year after hernia repair, computed tomography (CT) identified a mass lesion between the abdominal wall and the mesh, suggesting the possibility of peritoneal recurrence of rectal cancer. The accumulation of fluorodeoxyglucose (FDG) was evident via positron emission tomography-CT (PET-CT), while tumor marker levels were within the normal range. On laparotomy, the small intestine, abdominal wall, mesh, colon, and stoma were observed to be associated with the mass lesion, and en bloc resection was carried out. However, postoperative histopathological examination revealed an actinomyces infection without any cancerous cells.

**Conclusions:**

This case highlights the challenges faced by surgeons regarding preoperative diagnosis of actinomycosis, especially when it occurs after the resection of abdominal cancer. Also, this case reminds us of the importance of a histopathological examination for abdominal masses or nodules before starting chemotherapy.

## Introduction

*Actinomyces israelii* a gram-positive anaerobic rod-shaped bacterium, causes a chronic/subacute suppurative and granulomatous disease that is termed actinomycosis [1]. The most common pathogen of actinomycosis in humans is *Actinomyces israelii* [1, 2]. Actinomycetes are one of the resident microbiotas of the oral cavity, and the digestive and genital tract, and the most common sites of infection include the face, neck, chest, abdominal wall, and peritoneal cavity [1–4]. Thus, it is speculated that trauma, surgery, inflammation, and perforation of the gastrointestinal tract allow the pathogen to migrate into local tissues. The growth of the microbe is slow, and clinical manifestations of the infection appear gradually as the infection progresses [2, 5, 6], especially when host resistance is compromised. Based on literature review, it is claimed that the time from the initial event or trauma to the onset of actinomycosis varies widely, ranging from a few weeks to several years [4, 7]. Due to the ambiguous nature of the clinical features, diagnosing abdominal actinomycosis is difficult. Herein, we present a case of actinomycosis mimicking the peritoneal dissemination of previously resected rectal cancer.

## Case presentation

**Fig. 1:**
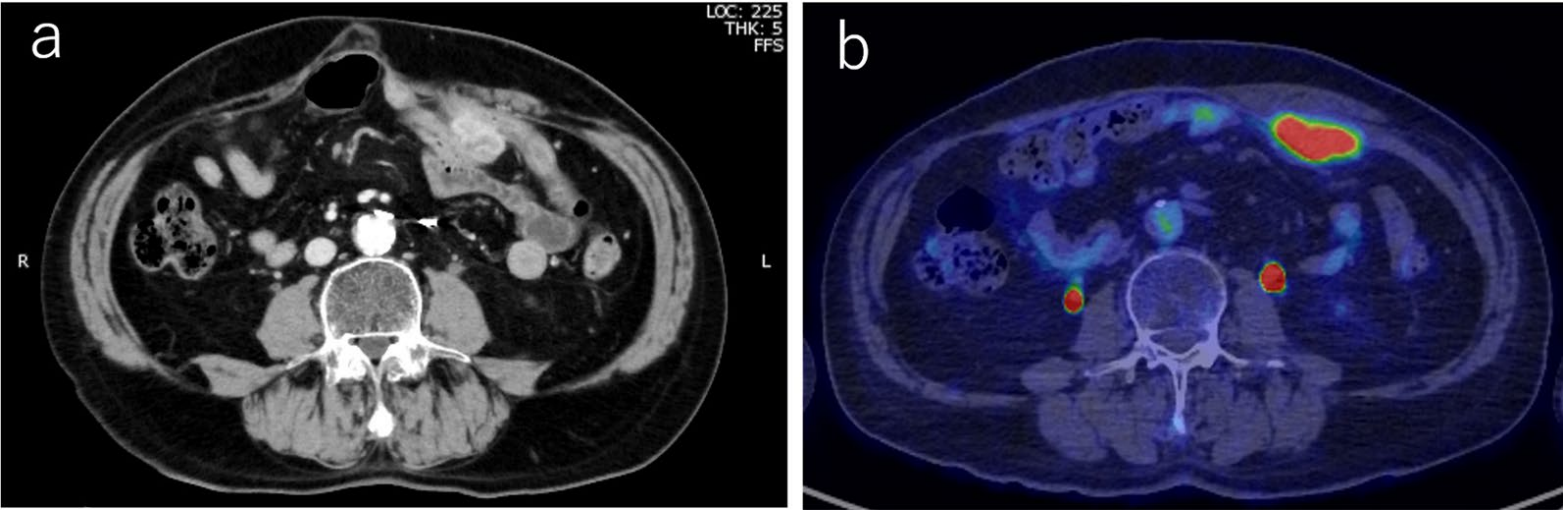
Radiological features of the case. **a** Abdominal computed tomography (CT) identified a mass near the stoma just below the abdominal wall. **b** There was no other nodule suggestive of peritoneal dissemination. The abdominal mass was removed along with the stoma, abdominal wall, mesh, and the associated portion of the small intestine

The patient is a 72-year-old man who previously underwent laparoscopy-assisted low anterior resection for rectal cancer. His medical history includes prostate cancer, hypertension, and spinal fracture, and he is an old stroke survivor. The surgical duration was 305 min with an estimated blood loss of 30 mL. Pathological diagnosis indicated Rb, Type 2, pPM0, pDM0, pT2, stage I, pR0, and pCurA. About 3 months later, stoma closure was performed. Eleven months post-initial surgery, the patient underwent S8 partial resection for metachronous liver metastasis, lasting 324 min with an estimated blood loss of 50 mL. Subsequently, resection of the remnant rectum with permanent stoma creation for local recurrence, including combined resection of the left seminal vesicle and pelvic nerve plexus, was performed laparoscopically, requiring 328 min. Four years after the initial surgery, the patient developed a parastomal hernia that was repaired using the Sugarbaker method under laparoscopy. The operative time for the hernia repair was 238 min. with minimal blood loss. One year after, abdominal enhanced computed tomography (CT) identified a mass near the stoma just below the abdominal wall that showed a substantial enhancement (Fig. 1a). The patient displayed no symptoms. His white blood cell count (6.1 × 10^3^/μL) and C-reactive protein level (0.12 mg/dL) were within the normal range. Tumor marker levels were also within the normal range: carcinoembryonic antigen (CEA) concentration was 1.3 ng/mL [normal range < 5.0 ng/mL] and carbohydrate antigen 19–9 (CA19-9) level was 14.7 U/mL [normal range < 37.0 ng/mL]. PET-CT imaging showed that the mass lesion displayed abnormal accumulation with a maximum standardized uptake value of 15.4, suggesting the peritoneal recurrence of rectal cancer (Fig. 1b).

**Fig. 2:**
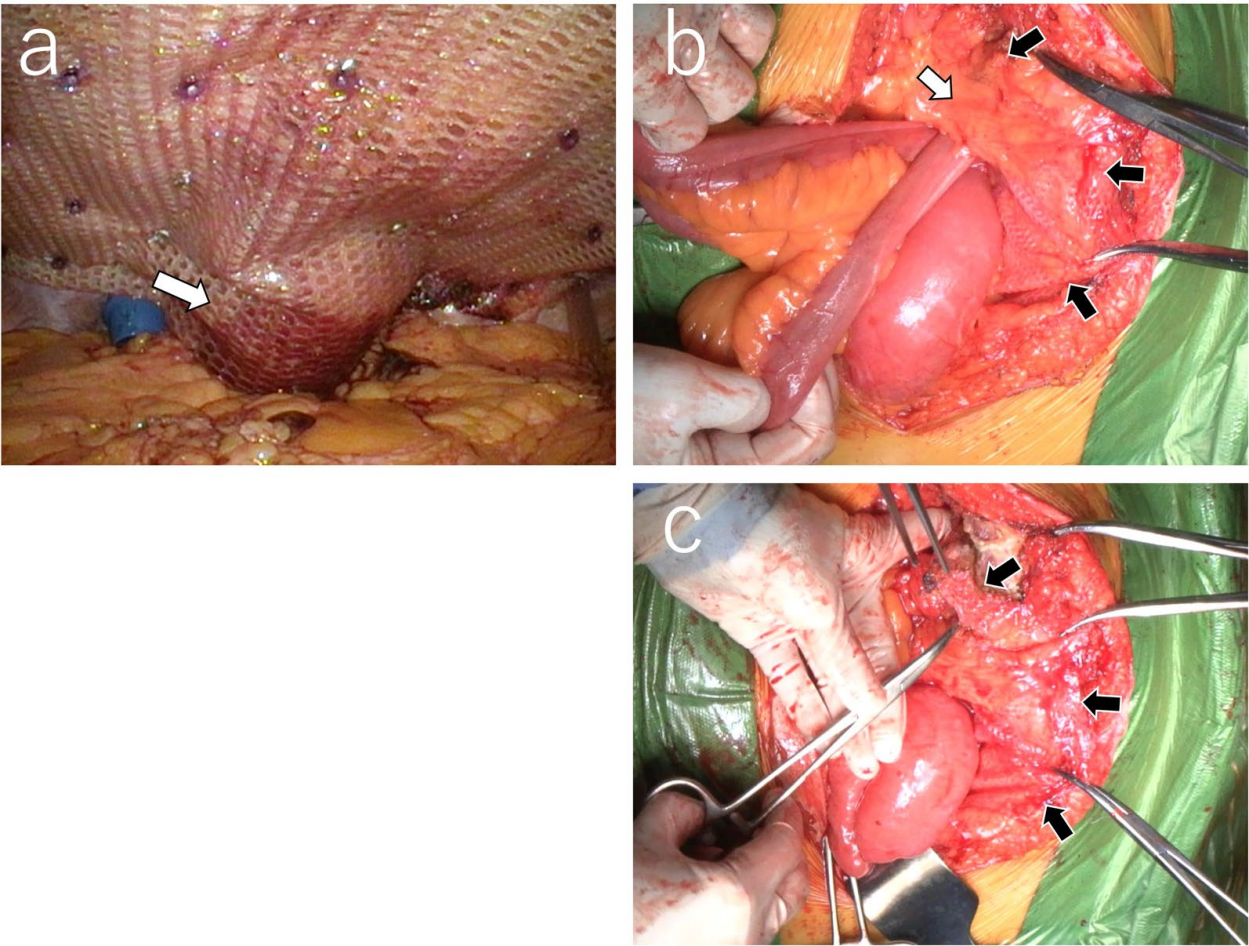
Surgical features. **a** Hernia repair using the Sugarbaker method was performed by placing a mesh on the dorsal side of the sigmoid and fixing it to the abdominal wall around the colostomy (The white arrow indicates the colon behind the mesh). **b** The small intestine is also involved in the development of lesions. The white arrow indicates the mass lesion covered by adipose tissue (omentum), while the black arrows indicate the mesh. **a** and **b** were rotated 180° from the original version). **c** The mesh (the black arrows) was removed from the abdominal cavity so that the mass lesion could be resected en bloc

The patient chose to undergo surgical resection of the mass lesion. Due to the sigmoid colon’s proximity to the colostomy, which was previously reinforced with a mesh using the Sugarbaker method (Fig. 2a), we anticipated that removing the mass lesion would require excising the mesh and performing partial resection of the colon, potentially involving the adjacent small intestine. Positioned near the ventral aspect of the abdominal wall, the mass presented challenges for laparoscopic surgery due to limited working space and restricted instrument mobility. Consequently, we opted for open surgery over laparoscopy.

**Fig. 3:**
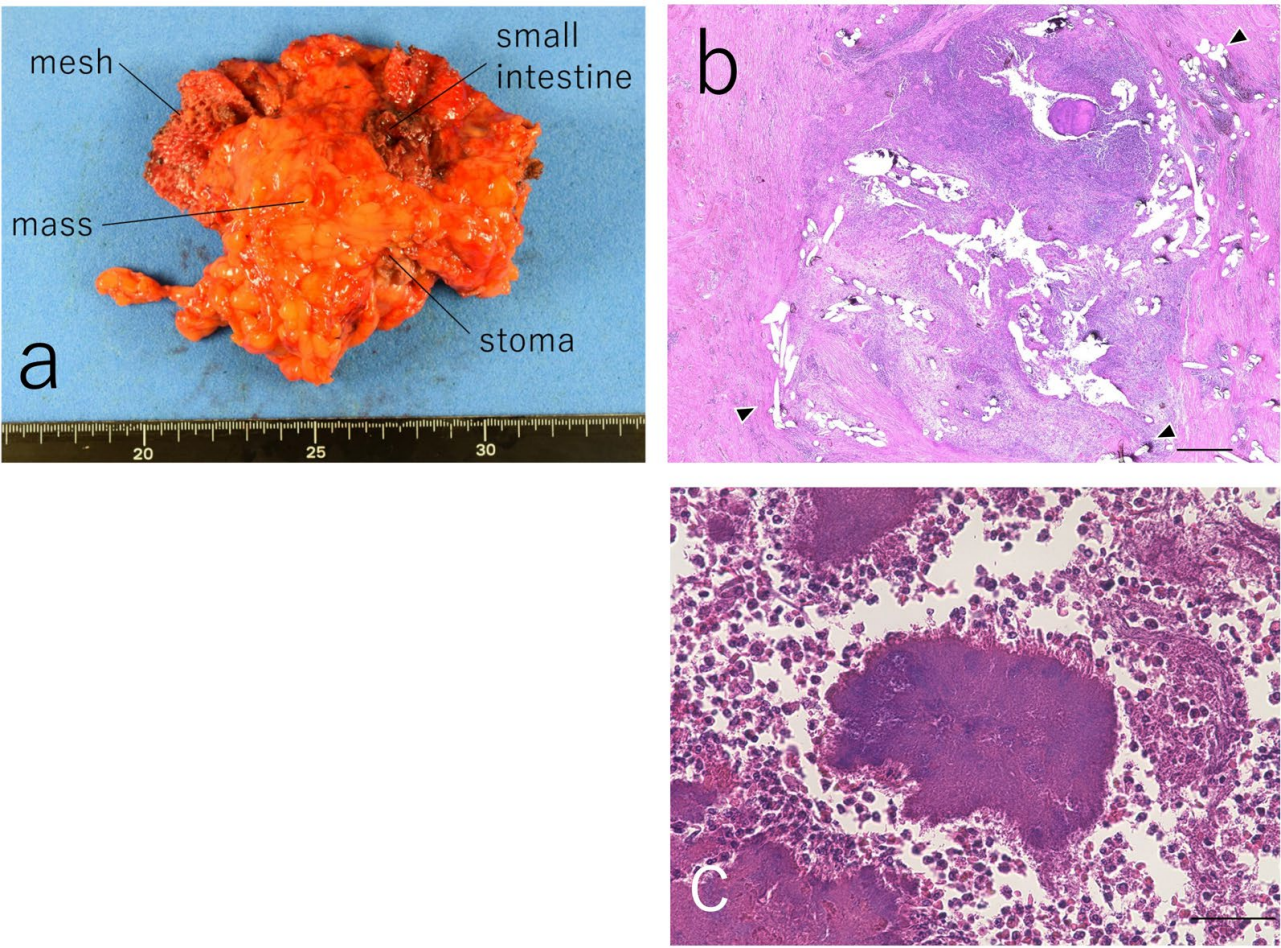
Macroscopic and microscopic features of the mass lesion. **a** The removed mass lesion is surrounded by the mesh, small intestine, colostomy, and adipose tissue. **b** Within the resected granulomatous tissue, fibrous structure of the mesh and multiple small holes (the black arrowheads) created by the mesh were observed. **c** Amorphous bacterial lesions consisting of hematoxylinophilic hyphae, markedly surrounded by neutrophils, were sparsely identified, occupying approximately 30% of the resected mass. Scale bar equals 50 μm. Images (**b**) and (**c**) were taken from different sections

On laparotomy, the mesh for hernia repair and the mass lesion were identified directly below the abdominal wall. However, there was no other nodule suggestive of peritoneal dissemination. Thus, the abdominal mass was removed along with the colostomy, abdominal wall, mesh, and the associated portion of the small intestine, because these structures could not be detached from the lesion (Figs. 2b, c and 3a).

On histopathological examination (Fig. 3b and c), no evidence of cancer cells was observed. Most of the mass was composed of granulomatous tissue, infiltrated inflammatory cells, and abscess formation. The fibrous structure of the mesh and artifacts (multiple small holes) created by the mesh were observed within the granulomatous tissue. Amorphous bacterial lesions consisting of hematoxylinophilic hyphae, markedly surrounded by neutrophils, were sparsely identified, occupying approximately 30% of the resected mass. Inside the granulomatous tissue, the fibrous structure of the mesh was observed, suggesting the Actinomycosis of the abdominal wall was finally diagnosed, and based on the surgical and pathological findings, the mesh was considered the primary site of infection. Postoperatively, the wound was partially opened due to wound infection. Blood tests showed a high inflammatory response, and cefmetazole was administered intravenously for 5 days. The patient was discharged on postoperative day 13. No further treatment has been provided so far.

## Discussion

*Actinomyces israelii*, a common pathogen causing actinomycosis in humans, is a gram-positive anaerobic bacterium resident in the oral cavity, gastrointestinal tract, and vagina. *Actinomyces israelii* enters the visceral organs when the intestinal mucosal barrier is disrupted by trauma, surgery, inflammation, or perforation, and the infection becomes evident when host resistance is compromised [1, 2]. An exceptional incidence involves actinomycosis at the abdominal wall without a history of surgery or trauma where hematogenous infection is the suspected cause [3, 8, 9]. Indwelling medical devices are related to the occurrence of actinomycosis. A retrospective study of 28 cases with abdominopelvic actinomycosis revealed that the majority of cases (17 out of 28) resulted from intra-uterine contraceptive devices, and two cases were caused by the presence of foreign bodies [10]. The authors of this retrospective study stated that the disease characteristics of 17 patients mimicked colon tumors or ovarian tumors, and only two cases had been preoperatively diagnosed as actinomycosis [10]. Notably, the formation of a mass lesion due to actinomycosis is not always solitary [11] nor limited to a single organ but can invade multiple organs [12]. These clinical features add to the difficulty of making a definitive diagnosis.

Regarding the cause of infection in this case, there are at least three conceivable opportunities for mesh infection. First, *Actinomyces israelii* could have entered the abdominal cavity during the initial and subsequent surgeries due to the disruption of the intestinal wall, remaining in the lower abdomen until it caused clinical symptoms. The considerable interval between potential microbial entry into the abdominal cavity and clinical manifestation may support this hypothesis [4, 7]. Second, *Actinomyces israelii* might have entered the abdominal cavity during the surgery for local recurrence, which required the creation of a colostomy. Furthermore, the microbes could have entered the space around the colostomy until complete wound healing prevented their migration. This explanation may be plausible as it aligns with the location of the mass. Third, organisms could have entered the bloodstream through injuries to the oral cavity or gastrointestinal tract, unrelated to abdominal surgeries [3, 8, 9]. The mucosa around the colostomy is often compromised due to inadequate stoma care, providing opportunities for organisms to enter the bloodstream. Skin erosion or ulceration around the stoma may yield similar outcomes. However, narrowing down these hypotheses to one in this case is challenging. This case underscores the importance of maintaining a clear surgical field (free from fecal or intestinal content contamination) and appropriate stoma care.

A bacterial culture or histopathologic examination can contribute to diagnosing actinomycosis in the abdomen [13]. However, the rate of diagnosis for actinomycosis from percutaneously collected tissue is low at least for two reasons. First, needle biopsy or aspiration may not reach the site of the bacterial infection because actinomycetes are covered with thick granulomatous tissue. Indeed, in our case, the actinomycetes formed only a tiny part of the whole mass. Second, the growth of *Actinomyces* is slow and requires an anaerobic, carbon dioxide-rich microenvironment [13], requiring repetitive sampling and/or more than 10 days of bacterial culture to conclude the negative results of culturing [1, 10]. In addition to these diagnostic difficulties, the results of blood tests also cause confusion. Although the disease is a type of infectious inflammation, white blood cell counts are often within normal range. C-reactive protein (CRP) levels are not always high, and in case these concentrations are high, the extent of the increase is slight [14, 15].

In the present case, a previous history of local recurrence of rectal cancer necessitated that peritoneal dissemination be considered first. Furthermore, a recurrence of tumors on the mesh has been reported previously [16, 17], where inflammation caused by the presence of a foreign body may have enhanced the implantation of cancer cells. The results of tumor marker levels may have reduced the possibility of recurrence in the present case. However, the level of carcinoembryonic antigen (CEA) does not consistently increase in the case of tumor recurrence: only one-third of blood test results in patients with tumor recurrence of colon cancer have demonstrated increased levels of CEA [18].

The nature of treatment after surgical resection remains controversial [2, 13]. A high recurrence rate (three out of four cases) of infection was reported among patients receiving short-term antibiotics [10]. Actinomycetes are, in general, susceptible to penicillin, tetracycline, clindamycin, and cefmetazole [1, 2]: a prolonged administration of these antibiotics is recommended on an empiric basis [6, 19, 20]. Further, lengthy administration of penicillin antibiotics may not always be necessary after the lesion has been extensively resected [13]. In our case, because the mesh that caused the infection was removed and other risk factors associated with a compromised immune system were absent, a long-term administration of antibiotics was not chosen. More than 16 months have passed since the operation without any signs of recurrence. An accumulation of additional cases is required to formulate guidelines that determine the treatment options after complete resection of the lesions.

## Conclusions

The present case highlighted the challenges involved in diagnosing actinomycosis, especially after abdominal surgery for malignancy. Although it is rare, actinomycosis should be included as an option in the differential diagnosis of an abdominal mass. The pathological examination of the abdominal mass is required when the diagnosis of tumor recurrence is not definitive.

## Data Availability

Not applicable.

## Abbreviations

CA19-9: Carbohydrate antigen 19–9
CEA: Carcinoembryonic antigen
CT: Computed tomography
CRP: C-reactive protein
FDG: Fluorodeoxyglucose
PET: Positron emission tomography
WBC: White blood cell count
